# Supplementary figures and images for: ‘This Is What the Colour Green Smells Like!’: Urban Forest Bathing Improved Adolescent Nature Connection and Wellbeing
Source: Int J Environ Res Public Health. 2022 Nov 24;19(23):15594. doi: 10.3390/ijerph192315594 (PMC9741002; doi:10.3390/ijerph192315594)

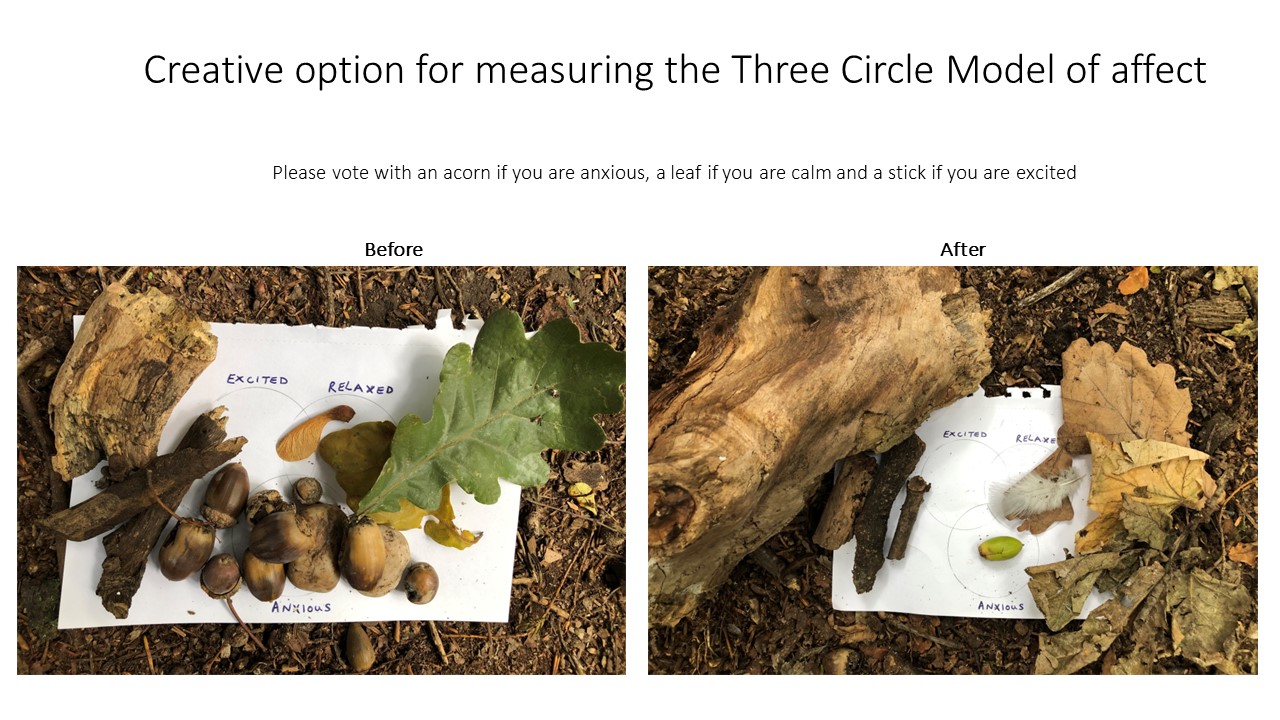

Supplement: Supplementary file 1 [file ijerph-19-15594-s001.zip › ijerph-1974832-supplementary.JPG]
